# DSCC_Net: Multi-Classification Deep Learning Models for Diagnosing of Skin Cancer Using Dermoscopic Images

**DOI:** 10.3390/cancers15072179

**Published:** 2023-04-06

**Authors:** Maryam Tahir, Ahmad Naeem, Hassaan Malik, Jawad Tanveer, Rizwan Ali Naqvi, Seung-Won Lee

**Affiliations:** 1 Department of Computer Science, National College of Business Administration & Economics Lahore, Multan Sub Campus, Multan 60000, Pakistan; 2 Department of Computer Science, University of Management and Technology, Lahore 54000, Pakistan; 3 Department of Computer Science and Engineering, Sejong University, Seoul 05006, Republic of Korea; 4 Department of Intelligent Mechatronics Engineering, Sejong University, Seoul 05006, Republic of Korea; 5 School of Medicine, Sungkyunkwan University, Suwon 16419, Republic of Korea

**Keywords:** skin cancer, melanoma, deep learning, transfer learning, CNN, dermoscopic images

## Abstract

**Simple Summary:**

This paper proposes a deep learning-based skin cancer classification network (DSCC_Net) that is based on a convolutional neural network (CNN) and implemented on three publicly available benchmark datasets (ISIC 2020, HAM10000, and DermIS). The proposed DSCC_Net obtained a 99.43% AUC, along with a 94.17% accuracy, a recall of 93.76%, a precision of 94.28%, and an F1-score of 93.93% in categorizing the four distinct types of skin cancer diseases. The accuracies of ResNet-152, Vgg-19, MobileNet, and Vgg-16, EfficientNet-B0, and Inception-V3 are 89.68%, 92.51%, 91.46%, 89.12%, 89.46%, and 91.82%, respectively. The results showed that the proposed DSCC_Net model performs better as compared to baseline models, thus offering significant support to dermatologists and health experts to diagnose skin cancer.

**Abstract:**

Skin cancer is one of the most lethal kinds of human illness. In the present state of the health care system, skin cancer identification is a time-consuming procedure and if it is not diagnosed initially then it can be threatening to human life. To attain a high prospect of complete recovery, early detection of skin cancer is crucial. In the last several years, the application of deep learning (DL) algorithms for the detection of skin cancer has grown in popularity. Based on a DL model, this work intended to build a multi-classification technique for diagnosing skin cancers such as melanoma (MEL), basal cell carcinoma (BCC), squamous cell carcinoma (SCC), and melanocytic nevi (MN). In this paper, we have proposed a novel model, a deep learning-based skin cancer classification network (DSCC_Net) that is based on a convolutional neural network (CNN), and evaluated it on three publicly available benchmark datasets (i.e., ISIC 2020, HAM10000, and DermIS). For the skin cancer diagnosis, the classification performance of the proposed DSCC_Net model is compared with six baseline deep networks, including ResNet-152, Vgg-16, Vgg-19, Inception-V3, EfficientNet-B0, and MobileNet. In addition, we used SMOTE Tomek to handle the minority classes issue that exists in this dataset. The proposed DSCC_Net obtained a 99.43% AUC, along with a 94.17%, accuracy, a recall of 93.76%, a precision of 94.28%, and an F1-score of 93.93% in categorizing the four distinct types of skin cancer diseases. The rates of accuracy for ResNet-152, Vgg-19, MobileNet, Vgg-16, EfficientNet-B0, and Inception-V3 are 89.32%, 91.68%, 92.51%, 91.12%, 89.46% and 91.82%, respectively. The results showed that our proposed DSCC_Net model performs better as compared to baseline models, thus offering significant support to dermatologists and health experts to diagnose skin cancer.

## 1. Introduction

The largest organ in the body is the skin, which saves the body from infection, heat, and UV light, but the serious threat to human life is cancer. The human body may harbor various kinds of cancer, and skin cancer is one of the deadliest and rapidly growing tumors. One in every three cancers diagnosed is skin cancer and, according to Skin Cancer Foundation Statistics, one in every five Americans will develop skin cancer in their lifetime [1,2,3,4]. In the USA, there are more than 3.5 million new cases that appear every year, and that number of cases is continuously increasing [3].

Many skin cancers begin in the upper layer of the skin. Skin cancer occurs when skin cells divide and expand in an uncontrolled way. New skin cells usually develop when old ones die or are damaged. When this process does not work correctly, cells grow quickly in an unordered way. This is why these cells are known as a tumor, which is in the form of a group of tissue [5,6]. It is caused by several factors, such as drinking alcohol, smoking, allergies, viruses, changing environments, and ultraviolet (UV) light exposure. Furthermore, skin cancer can also appear due to abnormal swellings on the body.

There are four different types of skin cancer: melanoma (MEL), melanocytic nevi (MN), basal cell carcinoma (BCC), and squamous cell carcinoma (SCC). The most dangerous category of cancer is MEL, because it spreads quickly to other organs. It arrives from the skin cells that are called melanocytes. On the skin, melanocytes create dark pigments, and these are mostly black and brown, while some are red, purple, and pink [7]. A melanoma cell frequently spreads to another organ, such as the brain, liver, or lungs [8,9]. Due to melanoma cancer, 10,000 deaths occur annually in the United States [10]. If it is identified early, then melanoma can be treated as soon as possible. It is not more common than other kinds of skin cancer. Melanocytic nevi (MN) happen in a pigmented mole that varies in a variety of skin tone colors. It mostly occurs throughout childhood and the early years of adult life, because the number of moles on one’s body increases up until the 30 to 40 years of age. Basal cell carcinoma (BCC) is the most common type of skin cancer. These are round cells that are created in the lower portion of the epidermis and normally grow slowly. Approximately all BCC develops on areas of the body that have a lot of sun exposure, including the face, neck, head, ears, back, and shoulders. Rarely, this type of skin cancer migrates to other body areas, and forms due to the abnormal and uncontrolled growth of cells. It may occur as a small, flesh-colored, or white tumor that may bleed. Squamous cell carcinoma (SCC) comprises flat cells found in the upper portion of the epidermis. These cancer cells can arise when cells grow uncontrollably. It may occur as a hard red mark or open sore that may bleed easily. Although this type of skin cancer is not normally dangerous, SCC can be found in numerous areas because it is usually generated by sun exposure. Additionally, it may also develop on skin that has already been burned or harmed by chemicals.

Skin cancer detection is a challenging process, and there are many different ways in which doctors can find skin cancer. An experienced dermatologist uses a sequence of steps to make a diagnosis, beginning with the naked eye detection of abnormal tumors, followed by dermoscopy, which uses a magnifying lens to conduct an in-depth analysis of lesion patterns, and the final step is biopsy [11,12]. Before the development of dermoscopic pictures, most skilled dermatologists had a rate of success of only 60 percent in diagnosing skin cancer, but dermoscopic images raised success rates to between 75 percent and 84 percent [13]. Additionally, correct identification is unique and largely dependent on the skills of the clinician [14]. The manual diagnosis of skin disorders is extremely difficult and stressful for the patient [15]. Computer-aided detection systems support health professionals to evaluated data garnered from dermoscopy method in situations where there is a shortage of professional availability or diagnostic expertise [16,17].

Skin cancer is a huge problem that needs to be investigated as soon as possible. The majority of people do not visit their dermatologist on a regular basis, which causes a fatally delayed diagnosis. The diagnosis is a manual process that takes a lot of time and money. However, diagnosis improved due to machine learning, and this can be useful in various ways. Skin cancer classification has been worked out using machine learning techniques, such as the support vector machine (SVM) [18], the Naïve Bayes (NB) classifier [19], and decision trees (DT) [20]. Convolutional neural networks (CNN) have gained popularity in recent years due to their ability to perform automatic feature extraction [21,22,23,24], as well as their broad use in research [25,26,27,28]. They are used to detect cancerous cells more rapidly and effectively.

The mortality rates are rising to alarming levels, yet if patients are detected and treated promptly, their chances of surviving are better than 95% [29,30,31,32,33,34]. Thus, this motivates us to develop a model for the early diagnosis of skin cancer to save human lives. In this paper, we present a novel multi-classification model, called the deep learning-based skin cancer classification network (DSCC_Net), based on the CNN, that identifies the four types of skin cancer, MEL, MN, BCC, and SCC, from dermoscopic images. Most of the research studies [29,30,31,32,33] have indicated great performance in binary classification, i.e., differentiating between benign and malignant skin cancer. However, no evidence has been found that uses the DL models for the classification of the skin cancers MEL, BCC, MN, and SCC. Additionally, DSCC_Net iwas also compared with six baseline classifiers: Vgg-19, Vgg-16, ResNet-152, EfficientNet-B0, Inception-V3, and MobileNet. The major contributions of this study are presented below:

- The novel proposed DSCC_Net model is designed to identify four different types of skin cancer. The proposed model has the capability of extracting dominant features from dermoscopy images that can assist in the accurate identification of the disease.
- In this study, we reduce the complexity of the model by decreasing the number of trainable parameters to obtain a significant classifier.
- The CNN model’s accuracy is compromised as a result of the problem of class imbalance in medical datasets. We overcome this issue by using an up-sampling technique, SMOTE Tomek, to obtain concoction samples of the image at each class to gain enhanced accuracy.
- The Grad-CAM heat-map technique is utilized to illustrate the visible features of skin cancer disease classification approaches.
- The proposed model achieved superior results, as compared to six baseline classifiers, Vgg-19, ResNet-152, Vgg-16, MobileNet, Inception-V3, and EfficientNet-B0, in terms of many evaluation metrics, i.e., accuracy, area under the curve (AUC), precision, recall, loss, and F1 score.
- Additionally, the proposed model also produced significant results as compared to the recent state-of-the-art classifiers.

This study is divided into the following section: Section 2 presents the literature review. Materials and methods are discussed in Section 3. The experimental results and discussion are presented in Section 4. This study is concluded in Section 5. 

## 2. Literature Review

Extensive research has been conducted on the diagnosis of skin cancer to better assist medical professionals in the process of detecting the disease at an earlier stage. Recent research, on the other hand, has been focused on developing different artificial intelligence algorithms to automate the diagnosis of several types of skin cancer. Table 1 presents the summary of recent literature on skin cancer diagnosis using DL models. 

Keerthana et al. [35] classified dermoscopy images as either benign or malignant cancers using two new hybrid CNN models, including an SVM algorithm at the output layer. The parameters extracted by the initial CNN model and the second CNN model are combined and passed to the SVM classifier. The accuracy of the first hybrid model with DenseNet-201 and MobileNet was 88.02%, whereas the accuracy of the second hybrid model with DenseNet-201 and ResNet-50 was 87.43%. Deep spiking neural networks were applied by Qasim Gilani et al. [36] to a total of 3670 melanoma images and 3323 non-melanoma images taken from the ISIC 2019 dataset. Using the suggested spiking Vgg-13 model, they attained an 89.57% accuracy and 90.07% F1-score, which was greater than that acquired with Vgg-13 and AlexNet, with fewer trainable parameters. Using the HAM10000 dataset, Kousis et al. [37] established 11 CNN architectures for several skin lesion classifications. They also built a mobile android application, in which DenseNet-169 architecture was applied that was relatively light, which identified the skin lesion as benign or malignant. Finally, DenseNet-169 was the model that achieved the highest accuracy (92.25%) when compared to other models, e.g., ResNet-50, Vgg-16, Inception-V3, etc. The second-highest accuracy has been achieved by the DenseNet-121 model. In terms of mobile applications, DenseNet-169 attained 91.10% accuracy. To accurately differentiate between malignant and benign melanoma, Kaur et al. [29] suggested an automatic melanoma classifier that was based on a deep CNN. The main goal was to suggest a lightweight and less-complicated deep CNN than other techniques, in order to efficiently identify melanoma skin tumors. The ISIC datasets were used to obtain dermoscopic pictures for this study that included several cancer samples such as ISIC 2016, ISIC 2017 and ISIC 2020. In terms of the ISIC 2016, 2017 and 2020 datasets, the suggested deep CNN classifier acquired accuracy rates of 81.41 %, 88.23 %, and 90.42 %. 

Alwakid et al. [38] employed the CNN model and modified ResNet-50, which was applied to a HAM10000 dataset. This analysis used an uneven sample of skin cancer. Initially, the image’s quality was improved using ESRGAN, then the next step taken to tackle the problem of class imbalance was the use of augmenting data. They achieved the result by using the CNN and ResNet-50 models, which were 86% and 85.3% accurate, respectively. Aljohani et al. [39] used CNN to perform binary classification for the detection of melanoma skin tumors. They used the ISIC 2019 dataset to test various CNN architectures for this purpose. The results of the experiment showed that GoogleNet achieved the maximum level of performance on both the training and testing data, in which they obtained 74.91% and 76.08% accuracies. Rashid et al. [30] used MobileNet-V2 to present a deep transfer learning network for the classification of melanoma. The MobileNet-V2 was a deep CNN that distinguished between malignant and benign skin lesions. The performance of the suggested DL model had been analyzed using the dataset of ISIC 2020. To solve the class imbalance problem, different data augmentation strategies were used. Ali et al. [40] applied EfficientNets B0-B7 models to the HAM10000 dataset of dermatoscopic images. The dataset contained 10015 images associated with seven different types of skin cancer, such as actinic keratosis (AKIEC), dermatofibrosarcoma (DF), non-vascular (NV), BCC, MEL, benign keratosis (BKL) and vascular skin lesions (VASC). Among the eight models, the EfficientNet-B4 represented the greatest Top-1 and Top-2 accuracies. In this experiment, the EfficientNet-B4 model achieved an 87% F1 score and 87.91% Top-1 accuracy.

Shahin-Ali et al. [31] used a deep CNN model by using the HAM10000 dataset. This data contained 6705 benign images, 1113 malignant images, and 2197 unknown images of lesions. The proposed model attained the highest training and testing accuracies, with 93.16 %and 91.93%, respectively. Furthermore, they balanced the dataset for both classes, which increased the accuracy of categorization. On the same dataset, they also trained several transfer learning models, but the results were not better than their proposed model. Le et al. [44] introduced a transfer learning model that comprised ResNet-50 without the use of a preprocessing stage or manual selection of features. All layers of the pre-trained ResNet-50 were used for the training in Google Colab. Global average pooling and dropout layers were employed to reduce overfitting. The images of the dataset were divided into seven different categories and the proposed model attained 93% accuracy. Bajwa et al. [41] created an ensemble model through the use of ResNet-152, SE-ResNeXt-101, DenseNet-161, and NASNet, to classify seven types of skin cancer with 93% accuracy. The ensemble was a technique of ML that merges the results of various distinctive learners to improve classification performance. Nugroho et al. [42] used the HAM10000 dataset to create a custom CNN for skin cancer identification. They used a scaled image with a resolution of 90 × 120 pixels. They achieved an 80% accuracy for training and 78% accuracy for testing.

Bassi et al. [45] used a DL technique that included transfer learning and fine-tuning. They resized the dataset images with the resolution of 224 × 224 and used a fine-tuned Vgg-16 model. They attained an accuracy of 82.8 %. Moldovan et al. [43] used a technique that was based on DL and transfer learning, in which they applied the HAM10000 dataset. The classification model was created in Python, utilizing the PyTorch library and a two-step process for classifying images of skin cancer. The first step’s prediction model was 85.0% accurate, and the second step’s prediction model was 75.0% accurate. Using dermoscopic images, Çevik et al. [46] employed the VGGNET model that contained a powerful CNN model to identify seven various kinds of disease. Images that were 600 × 450 pixels in size were analyzed and resized to 400 × 300 pixels. Sklearn, Tensorflow and Keras machine learning packages all were used in this Python-coded application. They obtained a score of 85.62 percent accuracy. Hasan et al. [47] developed the CNN-based detecting system that used feature extraction techniques to extract features from dermoscopic pictures. During the testing phase, they obtained an accuracy of detection of 89.5 %. However, the detection accuracy was insufficient and needed to be improved. Furthermore, there was overfitting between the testing and training stages, which was a flaw in that study. Saba et al. [31] suggested a deep CNN that used three phases to detect skin lesions: first, the color modification was used to improve contrast; second, a CNN approach was applied to extract the borders of the lesion; third, transfer learning was applied to remove the deep features. While the strategy produced good results for some datasets, the outcomes varied depending on the dataset. 

Using the dataset of ISIC 2018, Majtner et al. [48] created an ensemble of GoogleNet and Vgg-16 models. The authors performed the data augmentation and normalized its color to build the ensemble approaches they offered. The accuracy of the suggested method was 80.1%. Alquran et al. [33] introduced an image-processing-based approach for detecting, extracting, and classifying tumors from dermoscopy pictures, which aided in the diagnosis of benign and melanoma skin cancer significantly. The SVM classifier’s results showed an accuracy of 92.1%. Lopez et al. [49] described a deep-learning-based strategy to handle the problem of identifying a dermoscopic image that included a skin tumor as malignant and benign, with a focus on the difficulty of skin cancer classification, especially initial melanoma detection. The proposed solution employed the transfer learning approach that was based on the VGGNet CNN architecture. The proposed method obtained an accuracy level of 81.3% in the ISIC dataset, according to encouraging testing results. A linear classifier was built by Kawahara et al. [50] using a dataset of 1300 pictures and features collected by CNN to detect skin cancer. The method does not need skin lesion segmentation or preprocessing. They conducted classifications of five and ten classes, and their respective accuracy rates were 85.8% and 81.9%. Codella et al. [51] employed sparse coding, SVM, and deep learning to obtain an accuracy of 93.1% when evaluating recorded photos from the ISIC. These images were represented by bkl, mel, and nv. Krishnaraj et al. [52] designed machine learning [53,54,55,56] classifiers that identified binary classes of cervical cancer, such as adenosquamous carcinoma and SCC. They collected the dataset at the University of California, Irvine (UCI) repository, and the Borderline-SMOTE approach was employed to balance the unbalanced data. They obtained 98% accuracy through this dataset. Imran et al. [57] proposed a model that was based on deep CNN by using different layers and filter sizes. They used three different publicly available datasets: ISIC-2017, ISIC-2018, and ISIC-2019. In the ISIC-2017 dataset, they employed 2750 images that consisted of three labels: MEL, BKL, and NV. The ISIC-2018 dataset contains seven labels, in which 10,015 images were used, whereas the ISIC-2019 dataset implemented eight labels that contain a total number of 25,331 images. The accuracy rate of the ISIC-2017 dataset was 93.47%, while 88.75% and 89.58% accuracies were achieved by ISIC-2018 and ISIC-2019, respectively.

According to the above literature, it is extremely clear that a need still exists for a model with the ability detect the four different types of skin cancer with greater accuracy than current modalities. Although [29,30,31,39,47,49] performed a binary class classification of skin cancer, many other researchers were not able to handle multiclass classification with more successful outcomes. For multiclass skin cancer detection, the previous methods proposed in [40,41,42,43,44,45,46,47,48] were also unsuccessful at attaining a greater accuracy. Automated skin cancer classification in dermoscopic images is a challenging task due to high intraclass variance and interclass visual similarity. Furthermore, the presence of external and inherent artifacts and contrast between the affected and normal skin make it extremely difficult for the multiclassification of skin cancers. The proposed method overcomes the existing challenges, and effectively classifies the lesion into the four primary classes of skin cancer, MEL, SCC, BCC, and MN, with high efficiency.

## 3. Materials and Methods

This section presents the experimental procedure used to analyze the performance of the proposed model, as well as six well-known deep CNN models, which include Vgg-19, ResNet-152, Vgg-16, MobileNet, Inception-V3, and EfficientNet-B0.

### 3.1. Proposed Study Flow for the Diagnosis of Skin Cancer

When skin cells are exposed to UV radiation, their DNA is altered, which disrupts the skin cell’s normal growth and results in skin cancer. To find skin cancer, researchers frequently use dermoscopic images. DL algorithms are applied to enhance the accuracy of the detection of skin cancers, such as MEL, BCC, MN, and SCC. Furthermore, if skin cancer is diagnosed in its initial phase, health professionals have a better opportunity to prevent the disease’s growth and start treatment on time. The medical field has changed significantly as a direct result of the application of artificial intelligence and image processing. At this time, image processing is employed for analysis in almost every area of the medical field [58,59,60]. The community of researchers plays a significant role in the development of intelligent automated systems for accurate and speedy evaluations, and contributes to daily improvements of these systems [61,62,63].

**Figure 1:**
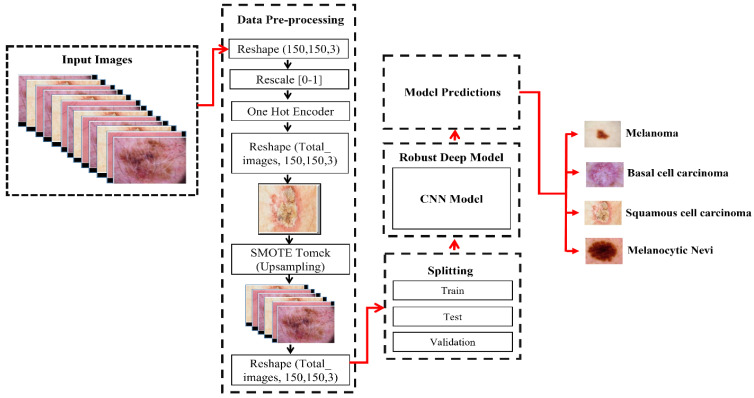
Workflow of the proposed DSCC_Net model.

For this study, we developed an automated system for the identification of skin cancers, called DSCC_Net. This system was trained and tested on images of four main categories of skin cancer: SCC, BCC, MN, and MEL. The input image’s size is fixed to a resolution of 150 × 150 pixels. In addition, the dataset was used according to the data normalization technique, in order to stop the model from being overfit. We also applied a technique called the synthetic minority oversampling technique (SMOTE) Tomek, in order to tackle the issue of an unequal distribution of datasets and to balance the number of samples within each class [64]. The skin cancer dataset is separated into three distinct categories that included training, testing, and validation sets. Furthermore, Figure 1 shows the work flow of the proposed DSCC_Net for skin cancer. In comparison to [65,66,67], the training parameter’s size is smaller. The experimental procedure was carried out for a maximum of 30 epochs. After completion of all the epochs, the proposed DSCC_Net achieved the expected level of accuracy throughout training and validation. The performance of the suggested method (DSCC_Net) was analyzed and was differentiated from that of six pre-trained classifiers: accuracy, loss, precision, recall, AUC, and F1-score. The Grad-CAM heat-map approach has been employed to illustrate the visible aspects of skin cancer that underline the qualities that affect its categorization. These characteristics have been used to highlight the aspects that lead to the diagnosis of skin cancer.

### 3.2. Dataset Description

**Figure 2:**
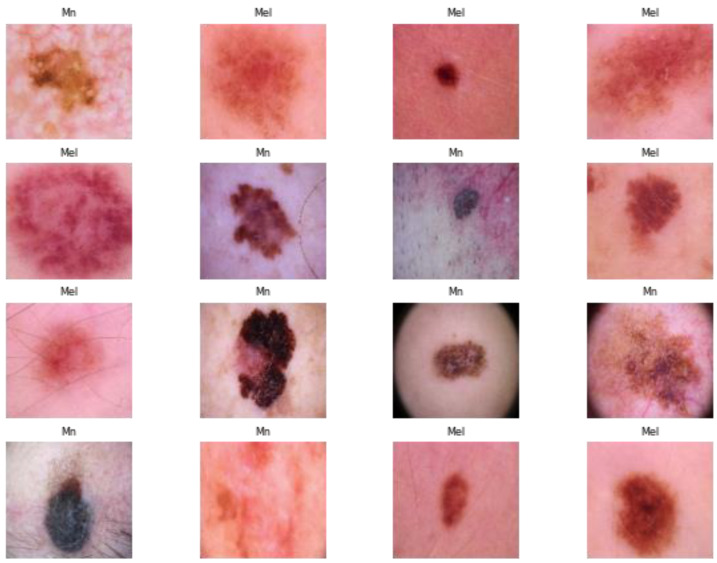
Original image samples of skin cancer extracted from three datasets.

On the internet, there are many freely accessible datasets of dermoscopy images. Because skin cancer is so common all around the world, this research focused on dermoscopy and photographic images of the disease. Images of four classes of skin cancer are shown in Figure 2. The proposed DSCC_Net was trained and tested on three datasets that were derived from three different resources. The ISIC-2020 Archive [68] is the world’s largest collection of dermoscopic images of skin lesions that are available to the general public. The images contained in this dataset were derived from a variety of different sources, because multiple institutes contributed patient data of various ages. There are 33,126 dermoscopic images, 579 images of malignant skin lesions, and 32,542 images of benign skin lesions. These pictures were taken from more than 2000 patients. We used 579 images of the melanoma class, and histopathology verified the diagnoses for all these images. The remaining images are all part of a benign class that was not considered for this research. Secondly, the HAM10000 database [69] includes 10,015 images that were produced by the International Skin Image Collaboration in 2018. Based on this information, this dataset consists of seven different data classes that identify the skin lesions. This database was developed by two different groups: Queensland University in Australia, and the ViDIR Group at the University of Vienna in Austria. In this dataset, we used 510 basal cell class images, 1107 melanoma class images, and 2007 melanocytic nevi class images. These dermoscopic images were taken from different populations, and the rest of the images were not considered in this study. Thirdly, dermis.net [70] is the most comprehensive online dermatology information source. It offers detailed images, differential diagnoses, and additional information on nearly all skin conditions.

### 3.3. Using SMOTE Tomek to Balance Dataset

**Figure 3:**
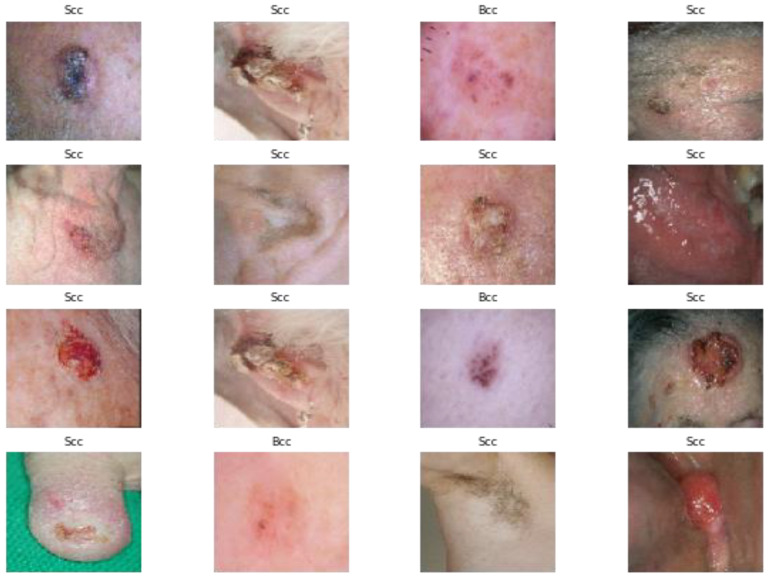
SMOTE Tomek generates samples of images to solve the class imbalance issue.

To resolve the issue of an unequal distribution of classes throughout the dataset, we applied the up-sampling method. In this method, we obtain fusion samples for each class by using the up-sampling algorithm SMOTE Tomek [64], as shown in Figure 3. This method is first applied to the class of observations belonging to minority classes. SMOTE is one of the most common and well-known oversampling methods used by data scientists to generate artificial minority points in the minority class examples. The aim was to combine SMOTE and Tomek techniques to improve the efficiency of dealing with the unbalanced class. Synthetic points are generated by SMOTE through the implementation of the KNN algorithm. The distribution of samples before the implementation of up-sampling is shown in Table 2.

### 3.4. Proposed Model

This section contains a complete description of the proposed DSCC_Net model.

#### 3.4.1. Structure of the Proposed DSCC_Net

**Figure 4:**
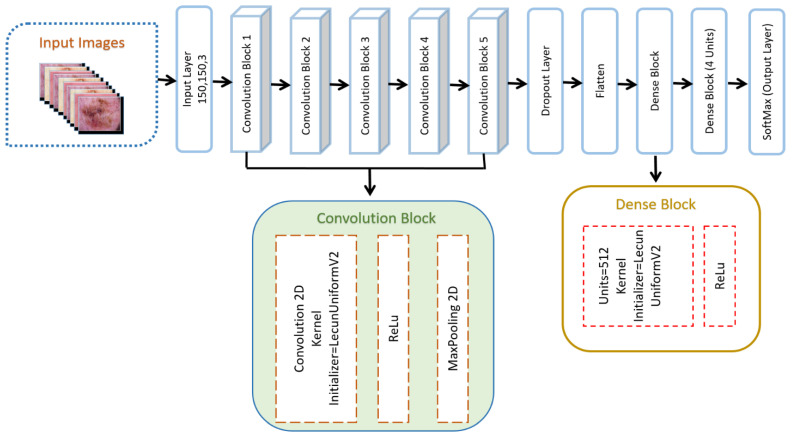
Architecture of proposed DSCC_Net used to classify skin cancer diseases.

The CNN structure is designed after the human brain’s biological anatomy, and is especially beneficial for applications of computer vision, such as object recognition, image segmentation, and face detection. According to the concept of translation or space invariance, a CNN can identify the same feature in multiple images regardless of where it occurs in the images [71,72,73]. In this study, we developed a robust DSCC_Net based on the CNN model to accurately classify skin cancer diseases. The DSCC_Net model consists of 5 convolutional blocks, and also includes a Rectified Linear Unit (ReLU) activation function, 1 dropout layer, 2 dense layers, and a softmax classification layer, as illustrated in Figure 4. Table 3 provides an overview of the dataset after the up-sampling technique, while a detailed explanation of the suggested DSCC_Net model for the categorization of skin cancer with the succeeding layers is presented in Table 4.

#### 3.4.2. Convolutional Blocks of CNN Model

The convolutional block is the fundamental building component of the presented work, and each convolutional block contains a convolutional 2D, a ReLU, and a pooling 2D with a max value. The initializer for the kernel layer LecunUniformV2 is created to assign layer kernel weights. The gradient-vanishing issue is solved by using the activation function of ReLU, which also simplifies the process for the network to understand and carry out its tasks in a timely way.

RGB channels are contained in the input image. Our model’s initial layer is known as the convolutional layer. This layer initiates the process by applying filters, also known as the kernel. The kernel’s size is dependent on two values, as illustrated in Equation (1).

(1) $$Filter\;Size\left(FS\right)=f_{w}\times f_{h}$$

where *f_w_* denotes the width of the filter and *f_h_* denotes the height of the filter. In our study, we set the size of the filter to 3, so Equation (1) becomes *FS* = 3 × 3. Feature identifiers are another name for these filters, and enable us to understand low-level visual aspects, such as edges and curves [74]. 

#### 3.4.3. Flattened Layer

**Figure 5:**
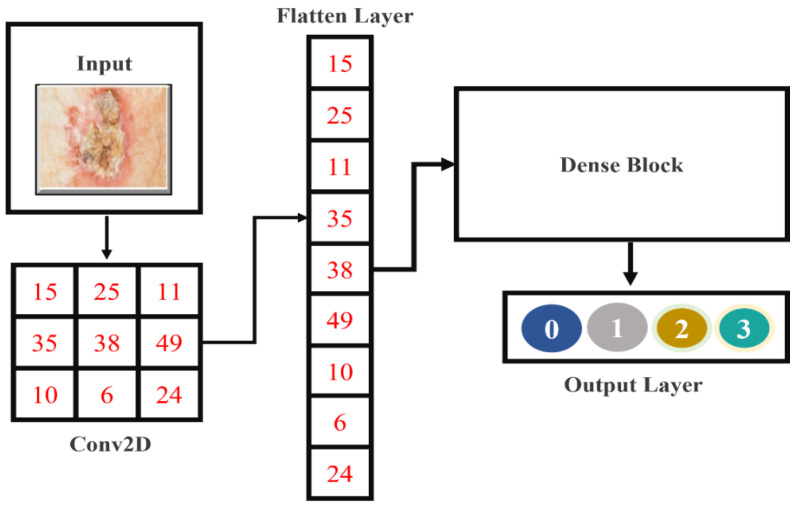
The fundamental structure of the flattened layer.

This layer is located among the convolution and dense layers. Tensor data types are used as inputs for the convolution layers, whereas dense layers demand a one-dimensional layout. So, the flattened layer was applied to translate the two-dimensional image representation into a one-dimensional input, which is presented in Figure 5.

#### 3.4.4. Dropout Layer

Our model utilized this layer with a dropout value of 0.2. This value was implemented in order to prevent the overfitting of our proposed DSCC_Net model [74]. The purpose of this layer was to switch units on and off to decrease the model’s training time and the complexity of the model. Consequently, the model learns the relevant features.

#### 3.4.5. Dense Block of Proposed DSCC_Net

In this research, we apply 2 dense blocks that consist of an activation function, which is explained in the following sections.

ReLU Function

Activation functions, which are mathematical processes, determine whether or not neural output should be passed on to the next layer. In general, they enable and disable the network nodes. Many activation functions are used in DL classifiers, but we applied ReLU due to its uncomplicated and time-saving computation. The activation of ReLU works by replacing all negative outcomes with zero. This activation function was used on the outputs of the convolutional layer.

Dense Layer

The dense layer accepts a single matrix as input and generates output according to its characteristics. In these layers, images are identified and given a class label. A dense layer with 4 neurons and a SoftMax activation function is responsible for generating the model’s final output, which classifies the image into one of the four skin cancer disease classes: MEL, BCC, SCC, and MN. SoftMax is applied after a few layers; this is a probability-based activation function in which the total amount of classes represents the number of neurons [69]. The total number of parameters is 1,149,524, which is split into two groups: 1,149,524 trainable parameters, and zero non-trainable parameters.

### 3.5. Model Evaluations

A confusion matrix was employed to check the performance of the model. Before training the model, the dataset was separated into training and test sets. The model was then evaluated using the test set. We applied a variety of metrics to evaluate the model’s performance. The following evaluation metrics (see Equations (2)–(5)) are widely employed to measure the effectiveness of the suggested DSCC_Net for skin cancer detection:

(2) $$Accuracy=\frac{TP+TN}{TP+FN+FP+TN}$$

(3) $$Precision=\frac{TP}{TP+FP}$$

(4) $$Recall=\frac{TP}{TP+FN}$$

(5) $$F1-score=2\times \frac{Precision\times Recall}{Precision+Recall}$$

## 4. Results and Discussion

We compare DSCC_Net to the most recently developed deep networks in the following section. The comparisons between the suggested DSCC_Net and six baseline deep networks are discussed in this section.

### 4.1. Experimental Setup

Keras was used to implement a total of seven models: six baseline models and the DSCC Net model. In addition, the programming of the approaches that are not directly connected to convolutional networks was achieved in Python. The experiment was achieved by using a computer running the Windows 10 operating system, equipped with an 11 GB NVIDIA GPU and 32 GB of RAM.

### 4.2. Accuracy Compared with Other Models

**Figure 6:**
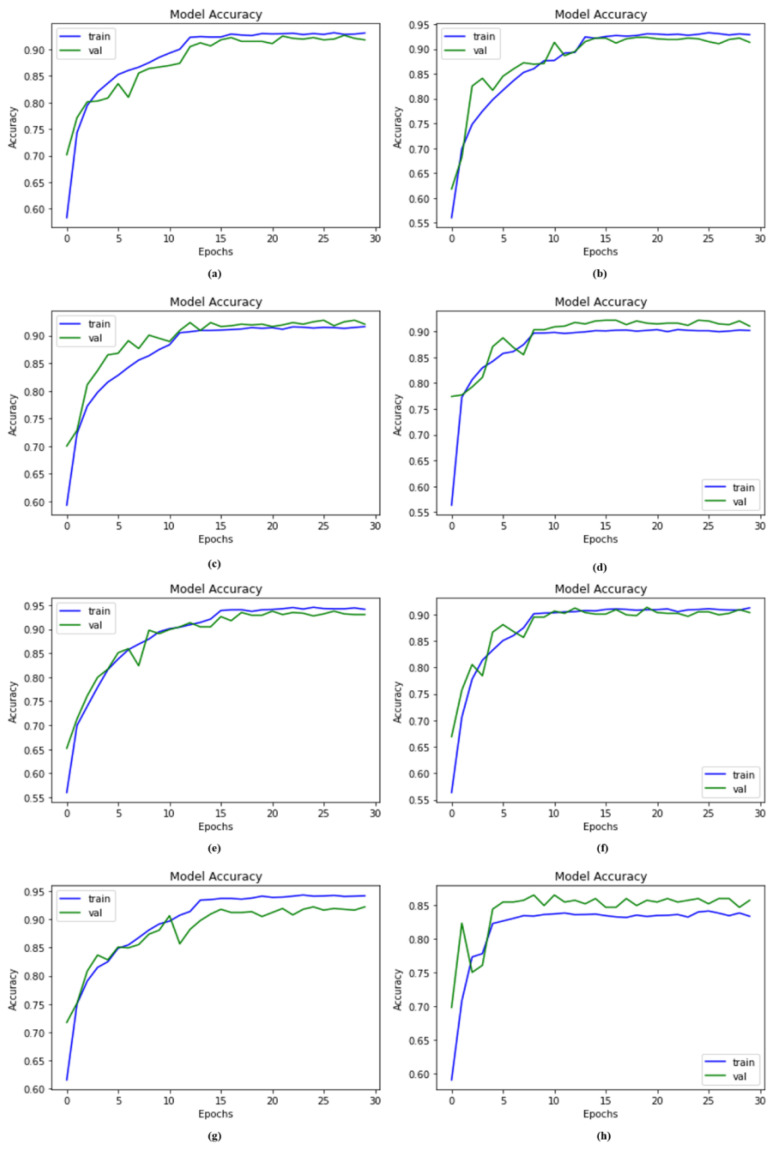
Remarkable accuracy improvement with or without SMOTE Tomek in the proposed model compared to other baseline deep networks; (**a**) Vgg-16, (**b**) Vgg-19, (**c**) EfficientNet-B0, (**d**) ResNet-152, (**e**) Inception-V3, (**f**) MobileNet, (**g**) Proposed Model with SMOTE Tomek, and (**h**) Proposed Model without SMOTE Tomek.

Using the same dataset and SMOTE Tomek, we compared our suggested and recent deep neural networks i.e., Vgg-19, ResNet-152, EfficientNet-B0, Vgg-16, Inception-V3, and MobileNet. We also compared the proposed DSCC_Net before applying the SMOTE Tomek. The system with SMOTE Tomek provides remarkable results for the proposed model. The obtained accuracies for the proposed DSCC_Net model with SMOTE Tomek, DSCC_Net without SMOTE Tomek, Vgg-16, ResNet-152, Vgg-19, MobileNet, EfficientNet-B0, and Inception-V3 were 94.17%, 83.20%, 91.12%, 89.32%, 91.68%, 92.51%, 89.46%, and 91.82%, respectively, as illustrated in Table 5. The significant improvement attained by the proposed DSCC_Net model, applying the SMOTE Tomek, is illustrated in Figure 6.

### 4.3. AUC Comparison with Other Models

**Figure 7:**
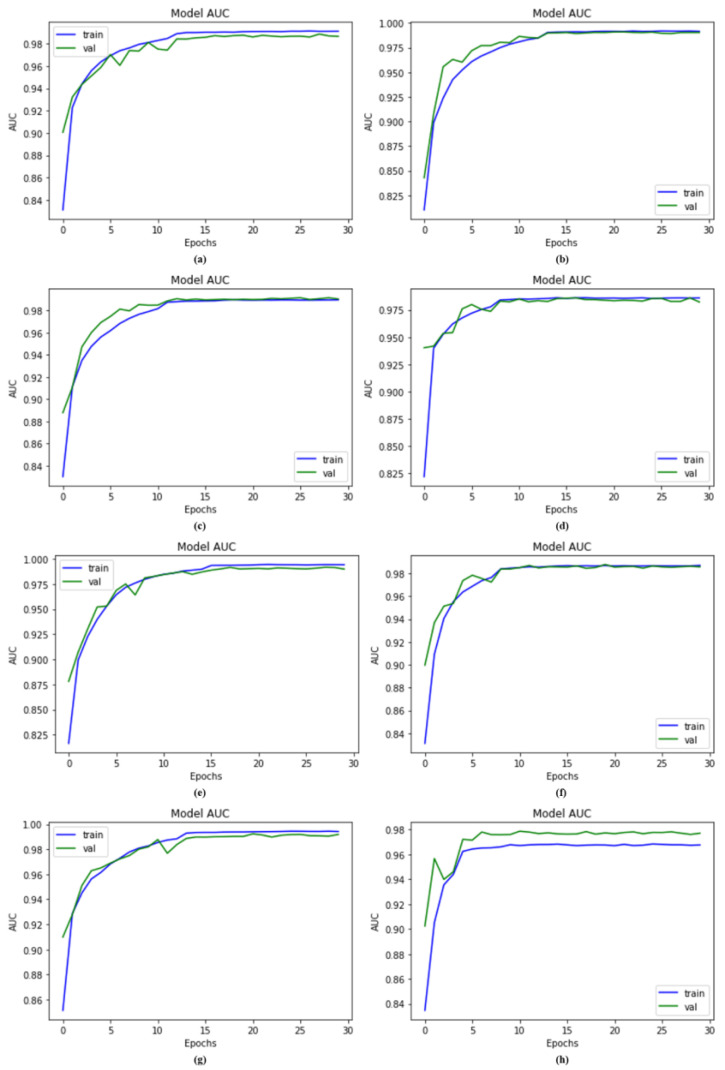
Results of the proposed DSCC_Net model with and without up-sampling; (**a**) Vgg-16, (**b**) Vgg-19, (**c**) EfficientNet-B0, (**d**) ResNet-152, (**e**) Inception-V3, (**f**) MobileNet, (**g**) Proposed Model with SMOTE Tomek, and (**h**) Proposed Model without SMOTE Tomek.

As discussed earlier in this research, our suggested model is a deep CNN-based DSCC_Net model containing multiple units that are particularly efficient in recognizing various skin cancer classifications. We compared DSCC_Net with five baseline deep networks to validate our proposed DSCC_Net model. Six baseline models, ResNet-152, Vgg-19, EfficientNet-B0, Vgg-16, MobileNet and Inception-V3, achieved the AUC values of 98.74%, 98.91%, 98.43%, 99.02%, 98.75% and 99.06% respectively. Figure 7 depicts that the proposed DSCC_Net with SMOTE Tomek and DSCC_Net without SMOTE Tomek achieved respective 99.43% and 96.65% AUC values when using the datasets. On the basis of the previous analysis, we conclud that the suggested model’s AUC results remain superior to those of other models.

### 4.4. Compared with Other Models Using Precision

**Figure 8:**
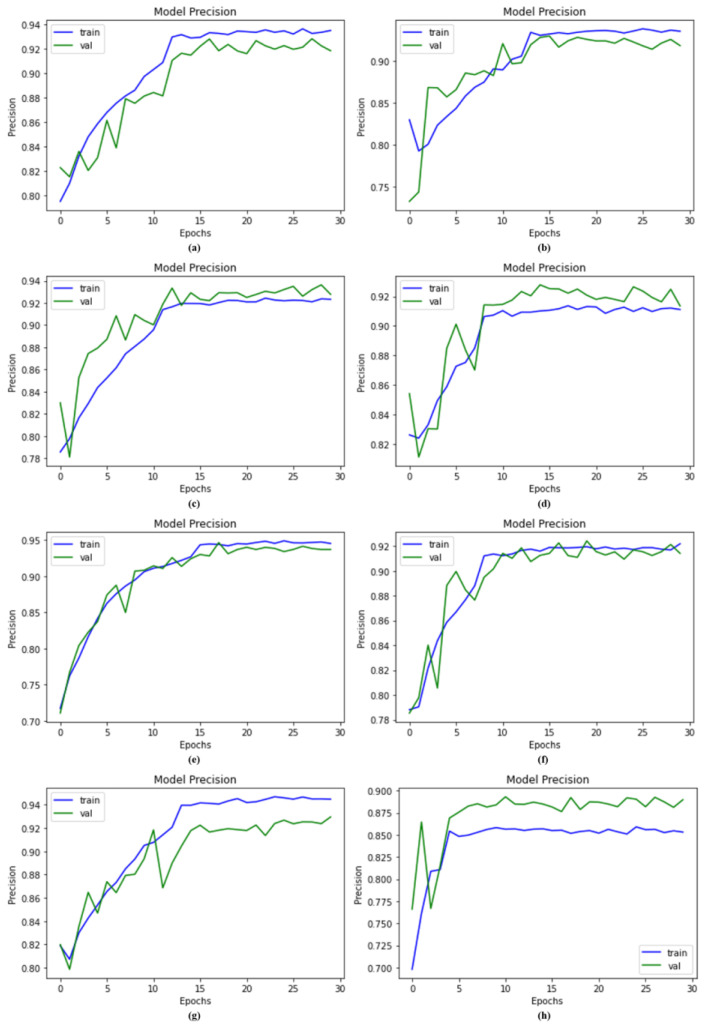
Precision results of the proposed model, DSCC_Net, and other baseline models; (**a**) Vgg-16, (**b**) Vgg-19, (**c**) EfficientNet-B0, (**d**) ResNet-152, (**e**) Inception-V3, (**f**) MobileNet, (**g**) Proposed Model with SMOTE Tomek, and (**h**) Proposed Model without SMOTE Tomek.

We examined our suggested and existing networks, such as ResNet-152, Vgg-19, Vgg-16, MobileNet, EfficientNet-B0, and Inception-V3, on the same dataset and balanced it using SMOTE Tomek. The system with SMOTE Tomek generated remarkable results for the proposed DSCC_Net. The proposed DSCC_Net with and without SMOTE Tomek attained precision values of 94.28% and 85.01%, but ResNet-152, Vgg-16, EfficientNet-B0, Vgg-19, Inception-V3, and MobileNet achieved precision values of 90.73%, 92.09%, 90.12%, 92.23%, 92.28%, and 92.95%, respectively. As a result of this analysis, we found that the suggested DSCC_Net ’s precision performance with SMOTE Tomek is superior and more consistent compared to recent models, as illustrated in Figure 8.

### 4.5. Compared of DSCC_Net against Other Models Using Recall

**Figure 9:**
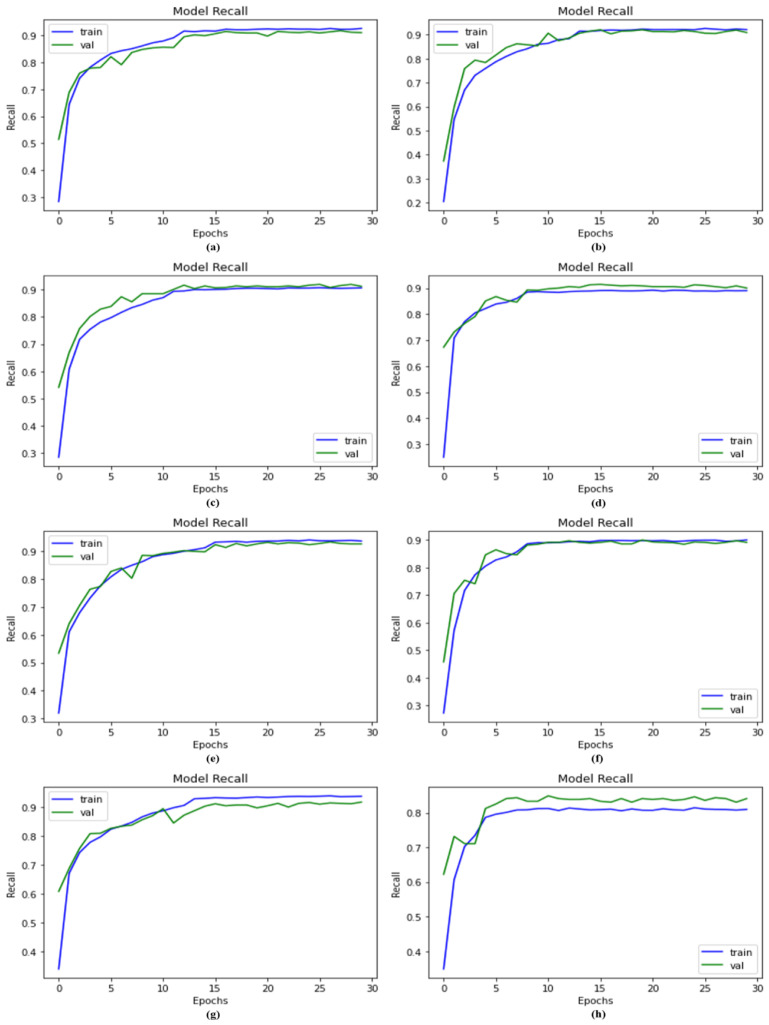
The recall analysis measures the proportion of true positive results correctly identified by a predictive model out of all actual positives; (**a**) Vgg-16, (**b**) Vgg-19, (**c**) EfficientNet-B0, (**d**) ResNet-152, (**e**) Inception-V3, (**f**) MobileNet, (**g**) Proposed Model with SMOTE Tomek, and (**h**) Proposed Model without SMOTE Tomek.

The model’s ability to identify positive samples was evaluated based on the recall metric. High recall values indicate that more positive samples were identified. The proposed DSCC_Net model was compared to other baseline deep networks using a recall curve, as illustrated in Figure 9. The proposed DSCC_Net with and without SMOTE Tomek, ResNet-152, EfficientNet-B0, Vgg-19, Inception-V3, Vgg-16, and MobileNet attained the recall values of 93.76%, 80.62%, 88.21%, 88.21%, 90.57%, 91.12%, 90.43% and 91.40%, respectively. As a result of the above explanation, the proposed method shows remarkable recall performance.

### 4.6. F1-Score Comparison with Recent Deep Model

**Figure 10:**
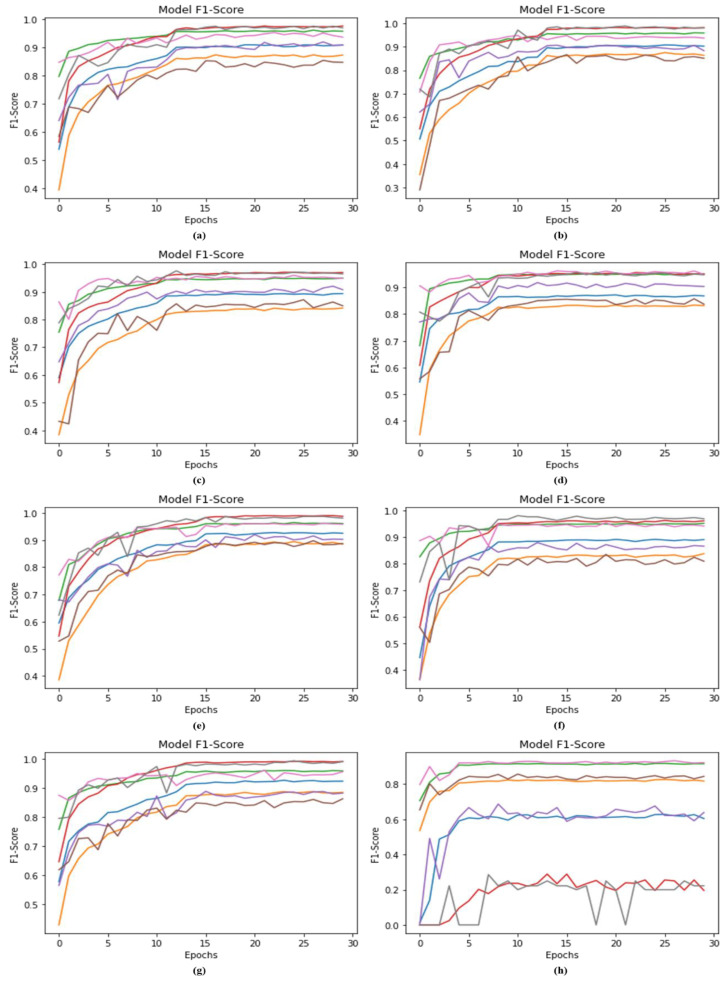
The value of the F1-score between the proposed model and six baseline models; (**a**) Vgg-16, (**b**) Vgg-19, (**c**) EfficientNet-B0, (**d**) ResNet-152, (**e**) Inception-V3, (**f**) MobileNet, (**g**) Proposed Model with SMOTE Tomek, and (**h**) Proposed Model without SMOTE Tomek.

The proposed DSCC_Net model with SMOTE Tomek and DSCC_Net without SMOTE Tomek achieved the F1-score values of 93.93% and 58.09%, respectively. Additionally, the six baseline models, ResNet-152, EfficientNet-B0, Vgg-19, Inception-V3, MobileNet and Vgg-16, attained the F1-score values of 89.27%, 89.31%, 91.71%, 91.76%, 92.17%, and 91.13%, respectively, as illustrated in Figure 10. The suggested DSCC_Net model attained the highest F1-score value with SMOTE Tomek shown in Figure 10.

### 4.7. Comparison of Proposed Model with Other Models Using Loss

**Figure 11:**
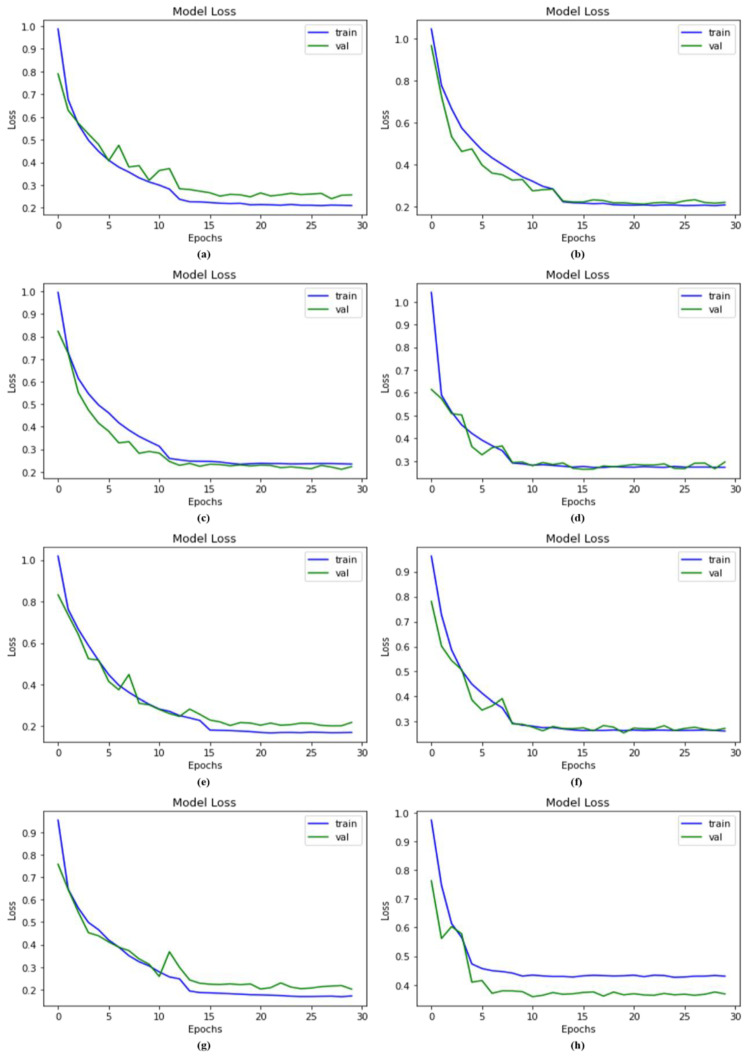
Loss value of the proposed DSCC_Net model and other baseline models; (**a**) Vgg-16, (**b**) Vgg-19, (**c**) EfficientNet-B0, (**d**) ResNet-152, (**e**) Inception-V3, (**f**) MobileNet, (**g**) Proposed Model with SMOTE Tomek, and (**h**) Proposed Model without SMOTE Tomek.

Loss functions are responsible for calculating the numerical difference between the predicted and actual values. In this study, a categorical cross-entropy method was utilized to calculate the loss. When the model was trained using up-sampled photos, however, the results were more remarkable. The proposed DSCC_Net model with and without SMOTE Tomek attained the loss values of 0.1677% and 0.4332%, whereas ResNet-152, EfficientNet-B0, Vgg-19, MobileNet, Vgg-16, and Inception-V3 achieved the loss values of 0.2613%, 0.2896%, 0.2353%, 0.2525%, 0.2279 and 0.2189, respectively. Figure 11 shows the major enhancement in the loss value of the suggested DSCC_Net model with SMOTE Tomek.

### 4.8. ROC Compared with Recent Model

**Figure 12:**
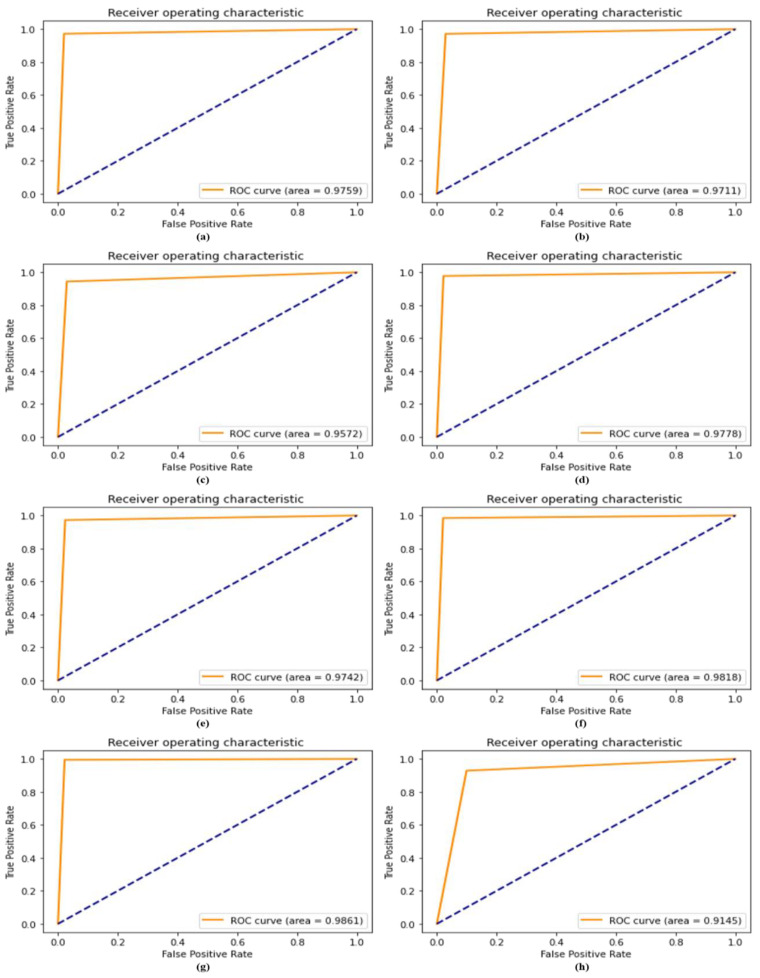
ROC curve comparing the performance of baseline models with the proposed DSCC_Net model; (**a**) Vgg-16, (**b**) Vgg-19, (**c**) EfficientNet-B0, (**d**) ResNet-152, (**e**) Inception-V3, (**f**) MobileNet, (**g**) Proposed Model with SMOTE Tomek, and (**h**) Proposed Model without SMOTE Tomek.

ROC iwa performed to evaluate the effectiveness of the diagnostic tests and, most specifically, the reliability of the binary or multi-classifier. A receiver operating characteristic (ROC) curve’s AUC is used to evaluate the effectiveness of a classifier; a higher AUC indicates that the classifier is more effective. Using the dataset, we evaluated the reliability of our proposed DSCC_Net model in terms of the ROC curve, both with and without SMOTE Tomek. This curve was used to compare the proposed DSCC_Net model, with and without SMOTE Tomek, to six baseline models on the same dataset. The suggested DSCC_Net with and without SMOTE Tomek, Vgg-19, Inception-V3, and MobileNet. ResNet-152, Vgg-16, and EfficientNet-B0 attained ROC values of 0.9861, 0.9145, 0.9711, 0.9742, 0.9818, 0.9778, 0.9759 and 0.9572, respectively, as shown in Figure 12. In the ROC curve, a significant enhancement of the suggested DSCC_Net model’s performance, with SMOTE Tomek, can be visible in Figure 12.

### 4.9. AU(ROC) Extension for Multi-Class Comparison against Recent Models 

**Figure 13:**
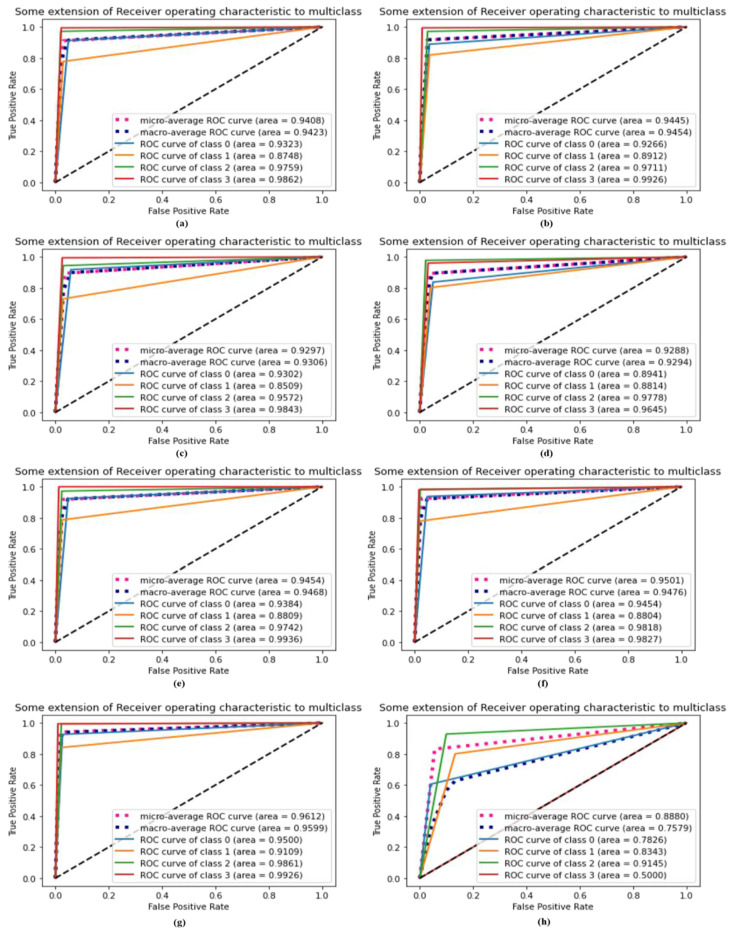
AU(ROC) curve evaluation with extension for the proposed model and other models; (**a**) Vgg-16, (**b**) Vgg-19, (**c**) EfficientNet-B0, (**d**) ResNet-152, (**e**) Inception-V3, (**f**) MobileNet, (**g**) Proposed Model with SMOTE Tomek, and (**h**) Proposed Model without SMOTE Tomek.

Figure 13 shows a comparison between the proposed DSCC_Net model and six baseline deep models using the ROC curve’s extension. After balancing the dataset by using the SMOTE Tomek technique, the suggested technique improved significantly as compared to the six models, which can be seen in Figure 13. The significant impact of the suggested DSCC_Net model was observed in terms of the AUC for both classes with and without SMOTE Tomek. The impacted classes include class 0 (BCC), class 1 (MEL), class 2 (MN), and class 3 (SCC). These enhancements in AUC provide evidence that the feature selection used by the DSCC_Net is accurate, and the SMOTE Tomek approach is also very useful.

### 4.10. Comparison of DSCC_Net with Six Models Using a Confusion Matrix

**Figure 14:**
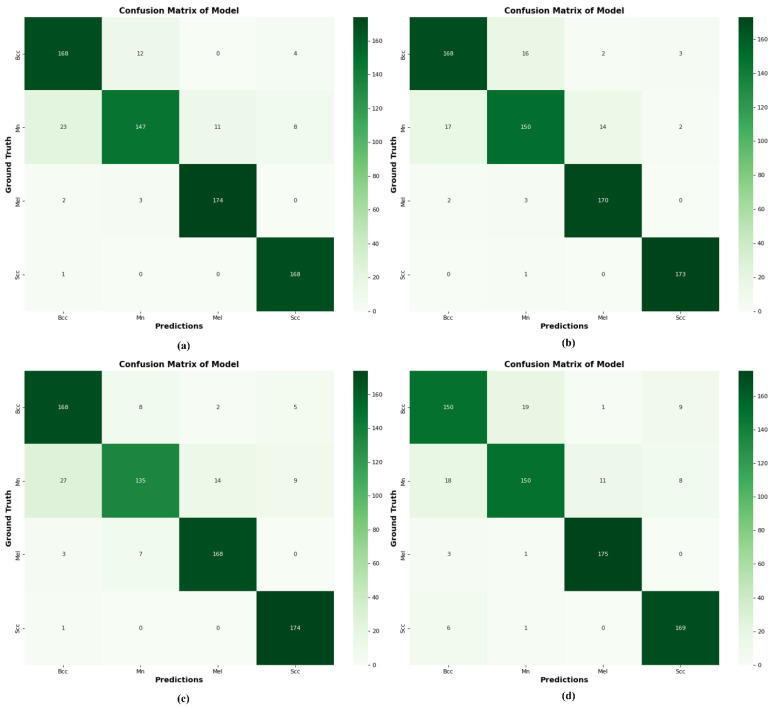

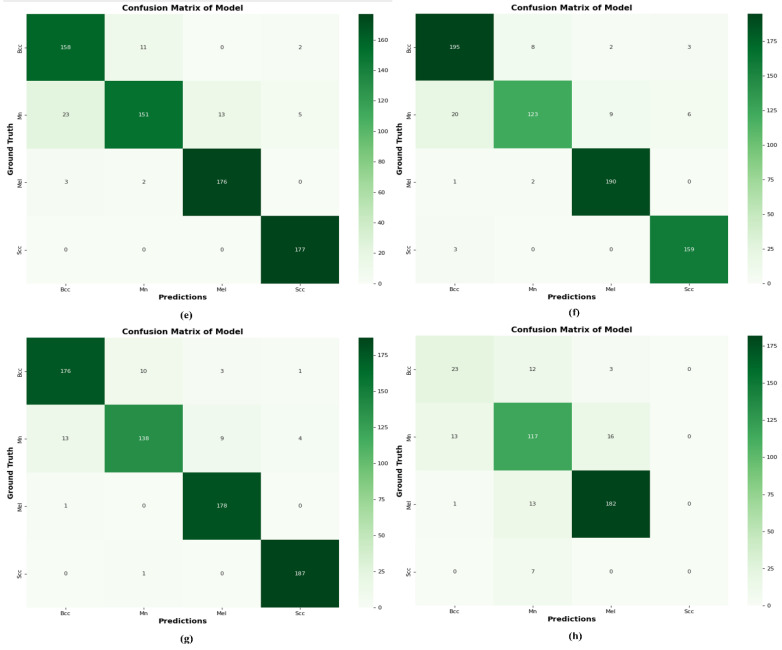
Using a confusion matrix to compare the proposed DSCC_Net with other deep networks; (**a**) Vgg-16, (**b**) Vgg-19, (**c**) EfficientNet-B0, (**d**) ResNet-152, (**e**) Inception-V3, (**f**) MobileNet, (**g**) Proposed Model with SMOTE Tomek, and (**h**) Proposed Model without SMOTE Tomek.

To validate our suggested DSCC_Net model with a confusion matrix, we compared it with six models. The use of SMOTE Tomek results in significant improvements for the DSCC_Net model, as presented in Figure 14.

**Figure 15:**
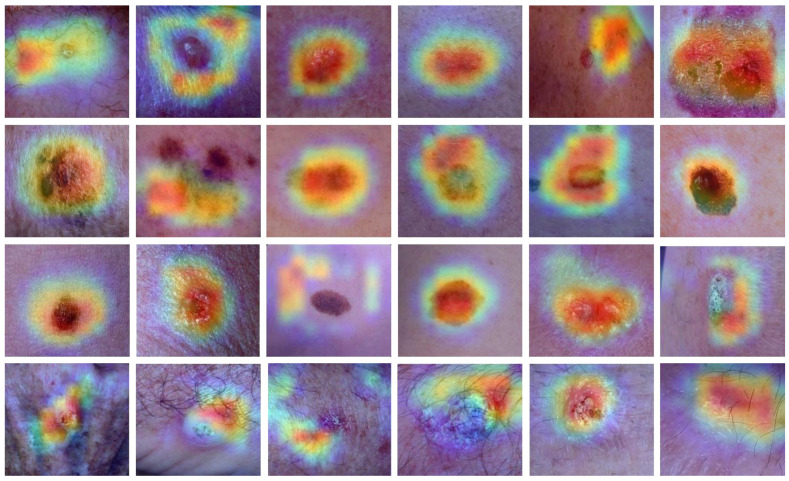
Grad-CAM evaluation of the proposed DSCC_Net model for skin cancer diseases.

The proposed method accurately classifies 176 images out of 190 total images in BCC cases, whereas it misclassifies 10 images as MN, 3 as MEL, and 1 as SCC. In MN classification, 138 MN images were correctly identified out of 164 total images, while 13 were misidentified as BCC, 9 as MEL images, and 4 as SCC images, as illustrated in Figure 14. The suggested method accurately identified 178 MEL images out of 179, whereas it misclassified one image as BCC. The DSCC_Net model correctly identified 187 SCC images out of 188 total images, while it misidentified one image as MN. In addition, we employed the Grad-CAM heatmap approach to visually represent the output of our suggested model. The objective of the heatmap is to show the relevant area of the skin that the model focuses on. Figure 15 illustrates the heatmap of the DSCC_Net model.

### 4.11. Comparison of the Proposed Model with State-Of-The-Art

In this section, we compare our proposed DSCC_Net model with previous modern studies [70,71,72,73,74,75,76]. Additionally, the proposed model is directly compared with the results reported in these [70,71,72,73,74,75,76] studies. Table 6 presents a comprehensive analysis of the proposed DSCC_Net model in terms of many performance evaluation metrics, such as accuracy, recall, F1-score, and precision, in comparison with the recent state-of-the-art studies.

### 4.12. Discussions

The identification and categorization of a wide range of skin cancers may be accomplished with the use of dermoscopy photographs [32,33,34,35]. Our method offers a full view of a particular site, which enables us to identify the disease, as well as interior areas that have been infected with it. Dermoscopy is the most reliable [41] and time-effective [52,53,54,55,56,57,58,59] approach for determining if a lesion is a BCC, MEL, SCC, or MN. A computerized diagnostic approach is required to identify BCC, MEL, SCC, and MN, since the number of confirmed cases of deadly skin cancer is continually growing [62]. Dermoscopy images might be able to automatically differentiate between those who have MEL and those who have other types of skin cancer, by using methods from the field of DL [64,65,66,67,68,69,70,71,72]. As a direct result of this, we developed a DSCC_Net model that is based on DL and is capable of accurately diagnosing a wide variety of skin diseases. These diseases include BCC, MEL, SCC, and MN, and the model enables dermatologists to begin treatment for their patients at an earlier stage. The three publicly available benchmark datasets (i.e., ISIC 2020, HAM10000, and DermIS) were used to evaluate the performance of the proposed DSCC_Net model. The results of the proposed model were compared with six baseline models: ResNet-152, Vgg-16, Vgg-19, Inception-V3, EfficientNet-B0, and MobileNet. The obtained image from datasets is imbalanced as discussed in Table 2. The imbalanced class of the images affected the performance of the model at the time of training [77,78,79,80,81,82]. To overcome these issues, we used the SMOTE Tomek technique to increase the numbers of images in the minority class of the datasets [49]. According to Figure 6, our proposed DSCC_Net model has received sufficient training on the four subtypes of skin cancer (BCC, MEL, SCC, and MN), and it can correctly identify occurrences of infection with these subtypes. Compared to the other six baseline skin cancer classifiers, our DSCC_Net model performs much better in classifying skin cancers, as discussed in Table 5. The DSCC_Net model using the SMOTE Tomek technique obtained an accuracy of 94.17%, regarding the categorization of dermoscopy pictures of BCC, MEL, SCC, and MN. Additionally, the DSCC_Net model used without SMOTE Tomek technique achieved an accuracy of 83.20%. On the other hand, the Vgg-16 model attained an accuracy of 91.12%. Similarly, the Vgg-19 and MobileNet models achieved an accuracy of 91.68% and 95.51%, respectively. The ResNet-152 model’s performance was poor in skin cancer classification as compared to all baseline models. Furthermore, we also provide the GRAD-CAM evaluation of the proposed DSCC_Net model for skin cancer disease classification as shown in Figure 15.

Table 6 presents the classification performance of the proposed DSCC_Net model with SOTA classifiers. Zhou et al. [70] proposed a DL model that achieved a classification accuracy of 0.92. Qasim et al. [71] designed a novel model, Vgg-13, for skin cancer identification. They achieved an accuracy of skin cancer detection of 89.57%. A ConvNet net model that focuses on the binary categorization of skin diseases was provided by Mijwil et al. [73]. This model was based on Inception-V3. By using this model, benign and malignant forms of skin cancer are distinguished. The multiclassification of skin lesions was performed by Afza et al. [74], by using 2D superpixels with ResNet-50, and they reached an accuracy of 85.50%. In addition, Khan et al. [75] attained a precision of 88.50% when performing the multiclassification of skin cancer. When compared to other approaches that are considered to be SOTA, the DSCC_Net model obtained an impressive accuracy of 94.71%.

## 5. Conclusions

In this study, the proposed DSCC_Net model, used for identifying the four forms of skin cancer (BCC, MEL, SCC, and MN), was developed and evaluated. Today, these skin cancer diseases are rapidly spreading and affect communities globally. Many deaths have occurred because of improper and slow testing procedures, limited facilities, and the lack of diagnosis of skin cancer at an early stage. Due to a large number of cases, a rapid and effective testing procedure is necessary. We proposed a DSCC_Net model to identify the four types of skin cancer diseases. Each convolutional block of the modified structure was generated using multiple layers and was applied in order to classify early-stage skin cancers. The SMOTE Tomek algorithm was used to generate samples that were used to solve dataset imbalance problems and to maintain a balance in the number of samples for each class. Grad-CAM displays a heat map of class activation to illustrate the operation of the CNN layer. Our proposed DSCC_Net model achieved 94.17% accuracy, 93.76% recall, 93.93% F1-score, 94.28% precision, and 99.42% AUC. So, it is concluded that DSCC_Net model can play a significant role as a supporting hand for the medical professional. The limitation of the study is that our proposed DSCC_Net model is suitable for only fair-skinned individuals. Individuals with dark skin were not considered in this study. The reason is that the publicly available datasets used in this work contain skin cancer images of fair-toned skin. In the future, we will combine blockchain and federated learning with a deep attention module to obtain more favorable results in classifying skin cancer, as well as skin infections.

## Figures and Tables

**Table 1:** Summary of the existing research studies for the diagnosis of skin cancer, using different machine learning and DL models.

Ref	Model	Type	Limitations	Dataset	Accuracy
[35]	Two hybrid CNN Models	Benign vs. Melanoma.	The classification accuracy of the model may be enhanced by using more advanced sampling techniques and data preparation.	ISBI 2016	88.02%
[36]	Spiking Vgg-13	Melanoma vs. non-Melanoma.	The model’s interpretability has to be improved.	ISIC 2019	89.57%
[37]	CNN, AlexNet, Vgg-16, Vgg-19	MEL, BCC, AKIEC, NV, BKL, DF, and VASC.	There is a limited selection of lightweight networks and hyperparameters for evaluation.	HAM10000	92.25%
[29]	Deep CNN	Malignant vs. Benign.	The model’s segmentation performance is fragile to occlusions in skin pictures, and it struggles with low-contrast skin disease images.	ISIC 2016, ISIC 2017, ISIC 2020	90.42%
[38]	CNN and ResNet-50	MEL, BCC, AKIEC, NV, BKL.	Different models and datasets call for various hyperparameter settings.	HAM10000	86%
[39]	DenseNet-201	MEL & non-MEL.	To further enhance the model’s generality, a more clinical dataset of skin-cancer cases is required.	ISIC 2019	76.08%
[30]	MobileNet-V2	Malignant & benign.	Overall accuracy drops when there is a large gap between the data domain and the target domain.	ISIC 2020	98.2%
[40]	EfficientNets B0-B7	MEL, BCC, AKIEC, NV, BKL, DF, and VASC.	The proposed model was trained and tested on an imbalanced dataset of skin cancer, and it affects the model performance.	HAM10000	87.91%
[31]	DCNN	Benign & malignant.	Due to the small sample size of the datasets used in this study, local optimizations may have been achieved.	HAM10000	91.93%
[41]	ResNet-152, SE-ResNeXt-101, DenseNet-161	MEL, BCC, AKIEC, NV, BKL, DF, and VASC.	The computational cost was significant, and the system did not take into account all possible skin cancers.	ISIC 2018	93%
[42]	CNN	MEL, BCC, AKIEC, NV, BKL, DF, and VASC.	Classification persists, however, because the model relies on a small quantity of training data and the hazy borders of skin disease pictures.	HAM10000	78%
[43]	DenseNet-121	MEL, BCC, and AKIEC.	Due to the lack of adversarial training on other skin cancer datasets, the method’s model remains vulnerable.	HAM10000	85%

**Table 2:** Image samples of skin cancer are distributed before up-sampling.

No. of Classes	Class Name	No. of Images
0	BCC	510
1	MEL	1686
2	MN	2007
3	SCC	97

**Table 3:** Image samples of the Skin Cancer dataset are distributed after up-sampling.

No. of Classes	Class Name	No. of Images
0	BCC	2035
1	MEL	1952
2	MN	2007
3	SCC	2018

**Table 4:** The total number of parameters utilized in the proposed DSCC_Net model.

Layer Type	Output Shape	Parameters
Input Layer	(None, 150, 150, 3)	0
Block01	(None, 150, 150, 8)	224
Block02	(None, 75, 75, 16)	1168
Block03	(None, 37, 37, 32)	4640
Block04	(None, 18, 18, 64)	18,496
Block05	(None, 9, 9, 128)	73,856
Dropout_1	(None, 4, 4, 128)	0
Flatten	(None, 2048)	0
Dense_1	(None, 512)	1,049,088
ReLu	(None, 512)	0
Dense_2	(None, 4)	2052
Output: SoftMax	(None, 4)	0
Total Parameters:		1,149,524
Trainable Parameters:		1,149,524
Non-Trainable Parameters:		0

**Table 5:** Performance of the DSCC_Net model compared with baseline algorithms.

Classifiers	Accuracy	Precision	Recall	F1-Score	AUC
Vgg-16	91.12%	92.09%	90.43%	91.13%	99.02%
Vgg-19	91.68%	92.23%	90.57%	91.71%	98.14%
MobileNet	92.51%	92.95%	91.40%	92.17%	98.75%
ResNet-152	89.32%	90.73%	88.21%	89.27%	98.74%
EfficientNet-B0	89.46%	90.21%	88.21%	89.31%	98.43%
Inception-V3	91.82%	92.28%	91.12%	91.76%	99.06%
Proposed Model (With SMOTE Tomek)	94.17%	94.28%	93.76%	93.93%	99.43%
Proposed Model (Without SMOTE Tomek)	83.20%	85.01%	80.62%	58.09%	96.65%

**Table 6:** Comparison of the DSCC_Net model with recent state-of-the-art studies.

Ref	Year	Model	Datasets	Accuracy	Recall	Precision	F1-Score
[70]	2023	CNN	ISIC-2017	92.00%	91.90%	91.65%	91.99%
[71]	2023	Vgg-13	ISIC-2019, Derm-IS	89.57%	90.70%	89.66%	89.65%
[72]	2023	Deep Belief Network	HAM-10000	93.00%	92.91%	92.45%	92.65%
[73]	2021	ConvNet	ISIC-2018, Derm-IS	86.90%	86.14%	87.47%	-
[74]	2022	2D superpixels + RCNN	HAM-10000	85.50%	83.40%	84.50%	85.30%
[75]	2021	ResNeXt101	ISIC-2019	88.50%	87.40%	88.10%	88.30%
[76]	2022	SCDNet	ISIC-2019	92.91%	92.18%	92.19%	92.18%
Ours	-	DSCC_Net with SMOTE Tomek	ISIC-2020, Derm-IS, HAM-10000	94.17%	94.28%	93.76%	93.93%

## Data Availability

Not applicable.
